# Evaluation of *in vitro* intrinsic radiosensitivity and characterization of five canine high-grade glioma cell lines

**DOI:** 10.3389/fvets.2023.1253074

**Published:** 2023-11-30

**Authors:** Benjamin Cartiaux, Alexandra Deviers, Caroline Delmas, Jérôme Abadie, Martí Pumarola Battle, Elizabeth Cohen-Jonathan Moyal, Giovanni Mogicato

**Affiliations:** ^1^INSERM UMR.1037-Cancer Research Center of Toulouse (CRCT), University Paul Sabatier Toulouse III, Toulouse, France; ^2^ToNIC, Toulouse NeuroImaging Center, Université de Toulouse, Inserm, UPS, ENVT, Toulouse, France; ^3^IUCT-oncopole, Toulouse, France; ^4^Department of Biology, Pathology and Food Sciences, Laboniris, Nantes, France; ^5^Unit of Murine and Comparative Pathology, Department of Animal Medicine and Surgery, Veterinary Faculty, Autonomous University of Barcelona, Barcelona, Spain

**Keywords:** glioma, canine, characterization, translational, radiation

## Abstract

Glioma is the most common primary brain tumor in dogs and predominantly affects brachycephalic breeds. Diagnosis relies on CT or MRI imaging, and the proposed treatments include surgical resection, chemotherapy, and radiotherapy depending on the tumor’s location. Canine glioma from domestic dogs could be used as a more powerful model to study radiotherapy for human glioma than the murine model. Indeed, (i) contrary to mice, immunocompetent dogs develop spontaneous glioma, (ii) the canine brain structure is closer to human than mice, and (iii) domestic dogs are exposed to the same environmental factors than humans. Moreover, imaging techniques and radiation therapy used in human medicine can be applied to dogs, facilitating the direct transposition of results. The objective of this study is to fully characterize 5 canine glioma cell lines and to evaluate their intrinsic radiosensitivity. Canine cell lines present numerous analogies between the data obtained during this study on different glioma cell lines in dogs. Cell morphology is identical, such as doubling time, clonality test and karyotype. Immunohistochemical study of surface proteins, directly on cell lines and after stereotaxic injection in mice also reveals close similarity. Radiosensitivity profile of canine glial cells present high profile of radioresistance.

## 1 Introduction

The increase of canine life expectancy, linked to improved nutrition, better vaccination, and enhanced veterinary care, is associated with a rise in age-related health conditions such as cancer. In fact, in the United States, more than 1 million cases of cancer are diagnosed in dogs each year (1) Among brain tumors in dogs, gliomas are the most common primary intracranial tumors, accounting for 36 to 70% of cases according to the literature (2). Brachycephalic dogs are the most commonly affected; among them, Boston Terriers, Boxers, and English and French Bulldogs are the most susceptible (3). It seems that genes predisposing to the development of gliomas exist and have been preserved through selective breeding of certain brachycephalic breeds. The majority of gliomas in dogs are localized in the cerebral hemispheres, preferably in the frontal lobe, the temporal lobe, and the parietal lobe (4). Clinical signs associated with gliomas in dogs are similar to those identified in humans. The most frequently encountered sign is the onset of seizures in nearly 50% of dogs. These seizures are a direct result of the mass effect exerted by the tumor on brain tissue, or they are secondary to peritumoral edema or neuroinflammation induced by the glioma. Dogs may also exhibit other nonspecific clinical signs such as lethargy, inappetence, or weight loss (5). The MRI appearance of canine gliomas tends to exhibit a similar pattern to what is observed in humans: they appear as hyperintense masses on T2 and iso- to hypo-intense on T1, with no contrast enhancement for low-grade gliomas and variable contrast enhancement for high-grade gliomas (6). The treatment of gliomas in dogs is diverse and relies on various methods such as surgery, chemotherapy, and radiotherapy. The latter approach has shown promising results in the treatment of gliomas in dogs, with an increase in median survival (7).

Pet dogs with spontaneously arising high-grade gliomas are becoming widely acknowledged as relevant translational models that could be used in addition to murine models. Indeed, the spontaneous oncogenesis of canine glioma is inherently associated with inter-tumor heterogeneity, genetic variability and anti-tumoral immune response that are lacking in immunocompromised xenograft mouse models. Canine high-grade gliomas closely resemble their human counterparts regarding oncogenic mechanisms (8–10), cancer stem cells (11–13), histopathological features (14), molecular alterations (15–19) and immune microenvironment (9, 20). Furthermore, the large size of their brain enables the use of the same RT devices and MRI scanners that those used in human medicine (21, 22). Taken all together, these data suggest that glioma-bearing dogs could be a well-suited pre-clinical model to predict the efficacy of radiosensitizers in human patients.

Even if the clinical response to RT is described in literature (21), to date there has been no published work evaluating the radiobiologic parameters of canine high-grade gliomas. These parameters are obtained *in vitro* by performing clonogenic assays which are considered as the gold standard for determining reproductive tumor cell death induced by ionizing radiation (IR). The surviving fraction at 2 Gy (SF2), the fraction of cells surviving a single 2 Gy dose of IR, and the mean inactivation dose (MID) are parameters that can be derived from clonogenic assays. SF2 and MID are useful tools to study the intrinsic radiosensitivity of cell lines and to predict *in vivo* tumor sensitivity to radiation (23–25). So far, four canine high-grade glioma cell lines have been put forward in the literature: J3T, J3T-Bg, SDT-3G, and G06A. The J3T cells were used to develop animal models of canine gliomas (26–28), and all of these cell lines have proven useful in understanding the mechanisms of oncogenesis in canine high-grade gliomas (8, 16, 17, 29), evaluating their sensitivity to chemotherapeutics (17, 30), targeted therapies (31–34) and viral vectors (27), as well as examining their miRNA profile in response to hypoxia (35). Recently, a new glioma cell line called Raffray, derived from a tumor originally classified as an anaplastic oligodendroglioma in a 7-year-old Boxer, was developed in a French laboratory (36).

In this context, the aim of the present study was to evaluate the intrinsic radiation sensitivity of these five canine high-grade glioma cell lines by performing clonogenic assays and examining the SF2 value and MID. As the tumor response to irradiation is dependent on the growth properties of cells and the proportion of tumorigenic cells, we have also evaluated the general and phenotypic characteristics of these cell lines and assessed their tumorigenicity.

## 2 Materials and methods

### 2.1 Cell culture

Canine cell lines were generously provided by Dr. Michael Berens (J3T), Dr. Peter Dickinson (J3T-Bg, SDT-3G, G06A) and Dr. Jérôme Abadie (Raffray). The J3T cell line was derived from an anaplastic astrocytoma in a 10 year-old male Boston Terrier (37). The J3T-Bg glioma cell line was derived from the canine J3T cell line following passage as a subcutaneous tumor through Biege-Nude-xid mice (16). The SDT3G and G06A cell lines were derived from glioblastomas in a 12 year-old male English Bulldog and a 2-year old female ovariohysterectomized Australian Shepherd, respectively (17). Raffray cell line was derived from an anaplastic oligodendroglioma in a 10-year-old female Boxer. All cell lines were grown in T-25 cm^2^ flasks with Dulbecco’s Modified Eagle Medium (Gibco™), supplemented with 10% fetal bovine serum, 1% penicillin/streptomycin (Gibco-BRL). Cells were maintained in an incubator at 37°C with 5% CO_2_.

### 2.2 Cell proliferation

Cells of each cell line were seeded at a concentration of 1×10^5^ cells per well in 6-well plates and maintained in supplemented DMEM (10% fetal bovine serum, 1% penicillin/streptomycin) at 37°C for 5 days. Every 24 h, two wells were trypsinized for 5 min at 37°C and the cells were counted using a Thomas cell counting chamber after a dilution 1: 1 with Trypan Blue. The average was carried out between the two wells. Replicates from three 6-well plates were scored each day. The doubling times of each cell line were computed using the “cell calculator++” tool (doubling-time. Com; Roth V., 2006).

### 2.3 Chromosome number

Metaphase cells were harvested by physical shaking and centrifuged at 500 g for 10 min, then they were resuspended in hypotonic solution of fetal calf serum (1:6) for 20 min, at 37°C. Cells were then centrifuged at 500 g for 10 min and fixed in ethanol: acetic acid (3,1) for at least an hour, at 4°C. Cells were spread on a slide and stained with 1% Giemsa solution to generate GTG banding. At least 20 metaphases were evaluated for each cell line to count the total number of chromosomes and the number of metacentric chromosomes per cell.

### 2.4 Orthotopic xenograft generation

Orthotopic canine high-grade xenografts were established in 8 week-old female nude mice (Janvier Labs) as previously described (38). Briefly, mice were anesthetized with intraperitoneal ketamine (100 mg/kg) and xylazine (10 mg/kg) and received a stereotaxically guided injection of 2.5 × 10^5^ cells suspended in 5 μL DMEM, using a Hamilton syringe. The injection was precisely located into the right forebrain (2 mm lateral and 1 mm anterior to the bregma at a 5 mm depth from the skull surface). The mice were observed for behavior changes suggestive of brain tumor (head tilting, circling, tremors) or general impairment of health (hypoactivity, decreased body weight of more than 20%, tachypnea, signs of pain such as aggressiveness, vocalization). When such changes were detected, they were humanely euthanized by anesthetic overdose by intraperitoneal injection of pentobarbital confirmed by cervical dislocation. Otherwise, mice were followed for behavior changes for a year. After euthanasia, brains were collected for subsequent histological and immunohistochemical analysis. All animals were treated according to the European Communities Council directive (2010/63/EU) regarding the care and use of animals, and all the experimental procedures were approved by the Institution animal ethics committee with authorization number 2018031614514282.

### 2.5 Histo-cyto-pathology and immunohistochemistry

Canine high-grade glioma cells cultured in supplemented DMEM were scraped off with a sterile scalpel and transferred into a 15 mL centrifuge tube. They were centrifuged at 400 rpm for 5 min, then resuspended in 10 mL phosphate-buffered saline (PBS) after removal of the supernatant. This operation was repeated one more time. The resulting cell suspension was centrifuged at 400 rpm for 5 min and resuspended in 1 mL of 70% ethanol after removal of the supernatant. The cell suspension was then transferred in a 1.5 mL microcentrifuge tube and centrifuged at 400 rpm for 5 min. Once the supernatant was discarded, the resulting pellet was resuspended in 50 μL of liquefied HistoGel™ (Thermo Scientific Richard-Allan Scientific, Kalamazoo, MI, United States) and the cell suspension was gently mixed to distribute cells evenly within the gel matrix prior to solidification (39). HistoGel-encapsulated cells were transferred in pathology cassettes, fixed in 10% neutral buffered formalin and processed as usual before embedding in paraffin. Excised brains were also fixed in 10% neutral buffered formalin. Paraffin-embedded excised brains and HistoGel-encapsulated cells were sectioned (5 μm) and stained with haematoxylin and eosin (HE) for microscopic evaluation. Immunohistochemistry (IHC) was performed in collaboration with the laboratory team of Dr. Martí Pumarola (12). Several IHC markers were used: CD133 as a stem cell marker, Olig2 protein and doublecortin (DCx) as glial and neuronal progenitor cell markers respectively; glial fibrillary acidic protein (GFAP) and vimentin (Vim) as mature astrocyte markers; S-100 protein as a mature oligodendroglial and astrocytic marker and βIII-tubulin and NeuN protein as mature neuron markers. Immunohistochemistry was performed in both HistoGel-encapsulated cells (DCx, GFAP, Vim, βIII-tubulin, and NeuN) and excised brains of mice which have developed tumors (Olig2, GFAP, Vim, S100, NeuN, and CD133). Olig2 expression was evaluated in all the xenograft samples and the other markers were evaluated only in two xenografts per tumor group. Procedures relative to antigen retrieval, endogenous peroxidase and non-specific binding blocking, antibody labeling and detection were performed as previously described (12). Primary antibodies, dilution and pre-treatment are summarized in Supplementary Table S1. The positive controls used for GFAP, Olig2, S-100, Vim, NeuN, and DCx immunostaining of xenogafts were samples of normal brain including grey and white matter, and skin for Vim immunostaining. Positive control for CD133 was adult canine healthy kidney tissue, because it is expressed by tubular epithelial cells. In all experiments, negative controls were obtained by omitting the primary antibody. A proportion score (corresponding to the percentage of positive neoplastic cells in each sample) and an intensity score (corresponding to the labeling intensity of positive neoplastic cells in each sample) were evaluated (12, 40). The percentage of positive neoplastic cells is obtained by taking the ratio of the number of labeled positive tumor cells to the total number of cells present in the field. Ten high-power fields were evaluated for each marker. An IHC score was then calculated (Table 1).

**Table 1:** Evaluation of the immunohistochemical score.

Proportion score (PS)	Percentage of positive neoplastic cells	Intensity score (IS)	Labeling intensity of positive neoplastic cells	Immunohistochemical score	PSxIS
0	<5%	0	No labeling	Negative	0
1	5–30%	1	Weak	+	From 1 to 3
2	30–60%	2	Mild	++	From 4 to 6
3	60–90%	3	Intense	+++	From 7 to 9
4	> 90%	−	−	++++	From 10 à 12

### 2.6 Radiosensitivity

Radiosensitivity was evaluated with a clonogenic survival assay. Canine high-grade glioma cell lines were dissociated, plated in T-25 cm^2^ flasks (1,500 cells/well) and irradiated at escalating doses (0, 1, 2, 4, 6, 8, and 10 Gy) with the Gamma-cell Exactor 40 (Nordion, Ottawa, ON, Canada), as previously described (41). Irradiated cells were subsequently incubated at 37°C for 15 days, to allow colony cell formation. Then, flasks were rinsed with 0.9% NaCl, fixed with FAA (Formalin – Acetic acid – Alcohol) fixative, and stained by 0.1% crystal violet. Each colony with more than 50 cells was considered as survivor. To calculate the surviving fraction at a given dose, the number of colonies is divided by the number of seeded cells and normalized to the plating efficiency of the not irradiated controls. The plating efficiency of not irradiated controls was determined as: (number of colonies formed/ number of cells inoculated) × 100. At least three independent experiments were carried out, from which a survival curve was drawn. The linear-quadratic (LQ) model is usually used to fit survival curves and to estimate several parameters of interest: (i) SF2 as the surviving fraction of tumor cells after a dose of 2Gy; (ii) MID (mean inactivation dose) as the average dose of the differential survival probability distribution; (iii) alpha (the linear component of the LQ model) that describes cell death due to lethal damage by a single incident; (iv) beta (the quadratic component of the LQ model) that represents cell death due to an accumulation of damage; and (v) the value of the alpha/beta ratio is inversely proportional to the sensitivity to fractionation of the biological effect considered (23, 42).

### 2.7 Statistical analysis

All results are represented as mean +/− standard deviation (SD). For statistical analysis, XLSTAT software (Data Analysis and Statistical Solution for Microsoft Excel, Addinsoft, Paris, France 2017). Kaplan–Meier estimate is used to evaluate the overall survival of mice with orthotopic xenografts of canine glioma cell lines. Log-rank test was assessed to analyze significant differences between the overall survival of different groups of cell lines. Results with *p* value <0.05 were considered statistically significant.

## 3 Results

### 3.1 General characteristics of the canine glioma cell lines

General characteristics of each cell line were established by assessing doubling time, plating efficiency, karyotype as well as cell morphology and protein expression profile (Table 2). Doubling times were in the same range (21 to 26 h) for all the cell lines except Raffray cells (56 h). The determination of plating efficiency, comprised between 16.4% (Raffray) and 23% (G06A), demonstrated that all glioma cell lines were able to give rise to colonies, which was the necessary condition to evaluate their radiosensitivity by *in vitro* clonogenic assay. Karyotyping revealed a number of chromosomes per cell slightly higher than the normal diploid chromosomal count for canines (78) and the presence of 1 to 2 abnormal metacentric chromosomes for all the cell lines but SDT3 which exhibited a chromosome number comprised between 59 and 61 and important chromosomal rearrangements.

**Figure 1:**
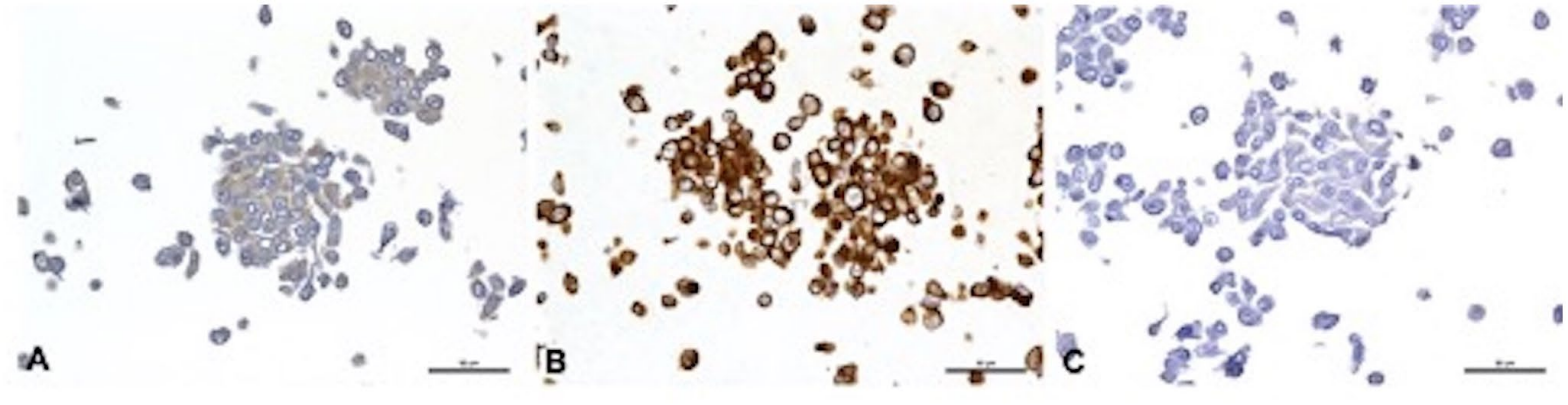
Immunohistochemical features of J3T-Bg cell line embedded in HistoGel™. **(A)** GFAP, weak cytoplasmic immunolabeling of 60 to 90% of cells. **(B)** Vimentin, strong cytoplasmic staining of more than 90% of cells. **(C)** NeuN, negative. Scale bar: 50 μm.

Cell morphology and protein expression profile were similar for J3T, J3T-Bg, G06A, and Raffray cells: they appeared mainly as spindle- to star-shaped cells with obvious cytonuclear atypia when observed in HE-stained slides and expressed the astrocytic markers GFAP (low immunohistochemical score) and Vimentin (high immunohistochemical score, Table 2). SDT-3 cells were smaller and more rounded, they showed a strong positive reaction to Vimentin but were negative to GFAP. Neuronal markers (DCx, NeuN, and βIII-tubulin) were negative for all cell lines. Taken all together, the immunohistochemical results were consistent with astrocytic cell lines. Immunohistochemical results obtained for the mature cell markers GFAP, Vimentin, and NeuN are displayed in Figure 1 for the cell line J3T-Bg.

**Table 2:** General genotypic and phenotypic characteristics of the canine high-grade glioma cell lines.

	J3T	J3T-Bg	SDT3	G06A	Raffray
DT (hours)	20.6	25.8	25.6	22.4	55.9
PE (%)	21.0	19.7	21.6	23.0	16.4
Number of chromosomes	78 to 82	78 to 81	59 to 61	78 to 82	76 to 81
Number of abnormal metacentric chromosomes	2	2	NE	1	2
GFAP	++	+	Neg	+	+
Vim	+++	++++	+++	++++	++++
DCx	Neg	Neg	Neg	Neg	Neg
βIII tubulin	Neg	Neg	Neg	Neg	Neg
NeuN	Neg	Neg	Neg	Neg	Neg

DT, doubling time; PE, plating efficiency; NE, not evaluated. The number of metacentric chromosomes for the SDT3 cell line was difficult to assess due to important chromosomal rearrangements.

### 3.2 Radiosensitivity

The values of dose response curve derived-parameters (SF2, α, β, α/β ratio, and MID) calculated for each cell line are summarized in Table 3. MID and SF2 were comparable for the 5 cell lines as exemplified by the low coefficient of variation (CV): the mean MID was 4.84 +/− 0.64 Gy (CV: 13%) and the mean SF2 was 0.84 +/− 0.08 (CV:10%) which is higher than the mean SF2 of cell lines derived from human tumors refractory to RT (43). The mean α was 0.027 Gy^−1^ +/− 0.029 (CV: 107%) and the mean α/β ratio was 0.836 +/−0.872 (CV: 104%).

**Table 3:** *In vitro* cell survival curve derived parameters of the canine high-grade glioma cell lines.

	J3T	J3T-Bg	SDT3	G06A	Raffray
α (Gy^−1^)	0.072	0.029	0.001	0.031	0.002
β (Gy^−2^)	0.033	0.032	0.025	0.030	0.030
α/β ratio (Gy)	2.182	0.906	0.040	1.033	0.067
SF2 (%)	73	91	93	85	80
MID (Gy)	4	4.6	5.7	4.7	5.2

### 3.3 Tumorigenicity

All the canine cell lines proved to be tumorigenic after orthotopic implantation in immunocompromised mice, with a tumor take rate varying from 25% for G06A cell line to 100% for J3T and J3T-Bg cell lines (Table 4). The number of mice used in experiments varies between 9 and 12, depending on the cell lines. To practice the surgical procedure, two unused mice from another study were added to the initial group of 10 mice from J3T cell lines. Unfortunately, before intracerebral injections, three mice passed away, with one from each group used for J3T-Bg, SDT3, and G06A cell lines.

**Table 4:** Tumorigenicity of the canine high-grade glioma cell lines.

	J3T	J3T-Bg	SDT3	G06A	Raffray
Number of mice with intracerebral injection of tumor cells	12	9	9	9	10
Number of brains submitted to microscopic examination	12	9	9	8*	8
Number of brains harboring a tumor	12	9	8	2	4
Tumor take rate	100%	100%	89%	25%	50%
Median survival (in days)	129	22	115	216	186

* Due to marked autolysis, three brains could not be analyzed (1 brain from the G06A group and two brains from the Raffray group).

**Figure 2:**
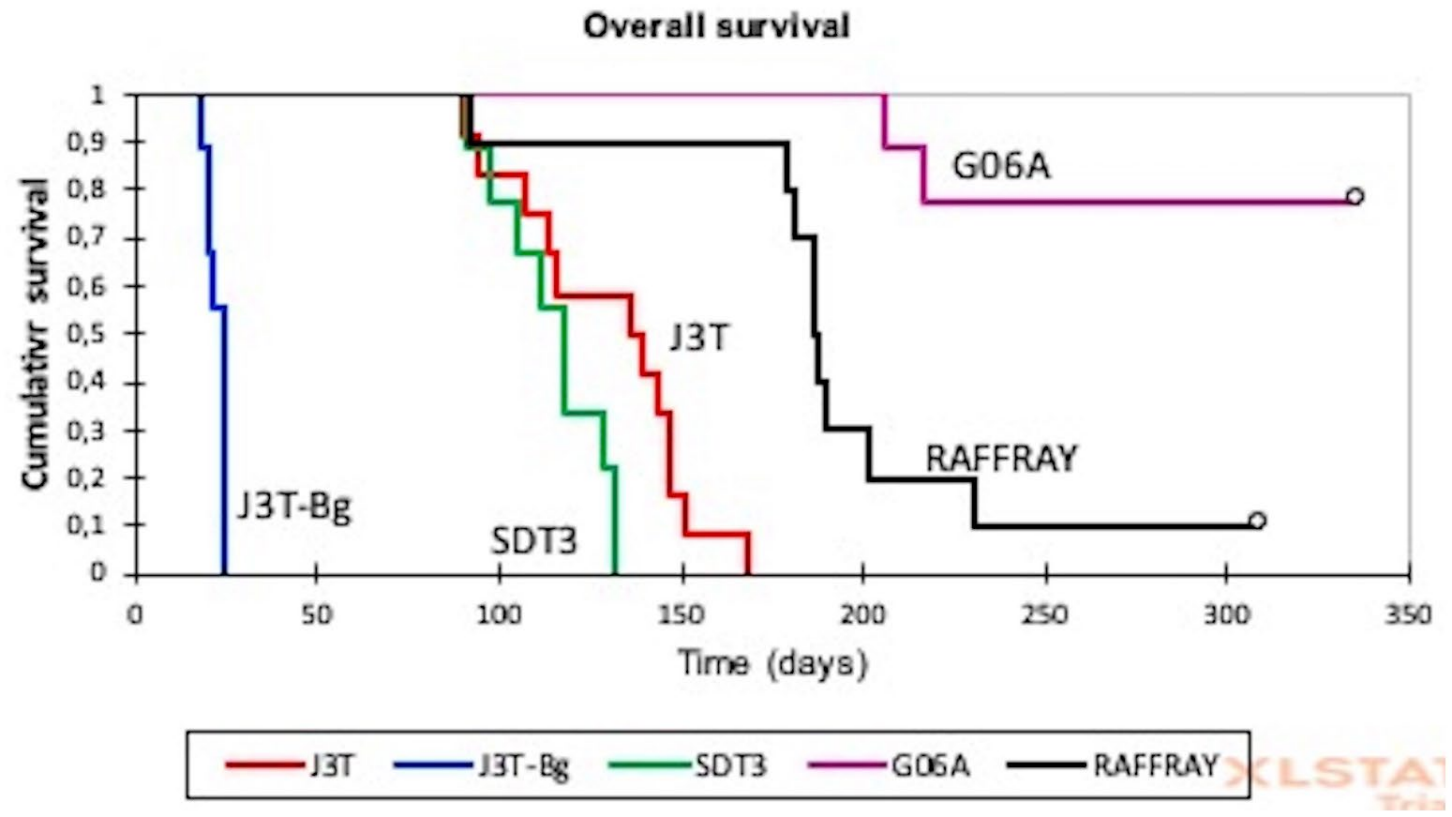
Kaplan–Meier analysis of overall survival in canine glioma-derived mouse orthotopic xenografts. The median survival of canine glioma-derived mouse orthotopic xenografts was significantly different depending on the cell line. The lowest median overall survival of 22 days (20–23) was observed for J3T-Bg, and the highest concerned G06A and Raffray cell lines with, respectively, 216 and 186 days. White circles represent surviving mice at the end of experiment.

The median survival of canine glioma-derived mouse orthotopic xenografts was significantly different depending on the cell line that was injected in the brain (Figure 2). The median overall survival was the highest for G06A (216 days, 95% CI: 212–218) and for Raffray-derived mouse orthotopic xenografts (186 days, 95% CI:162–210). In these two groups, mice were still alive at the endpoint of experiment (represented by white circles in Figure 2). The lowest median survival was 22 days (20–23) for J3T-Bg.

**Figure 3:**
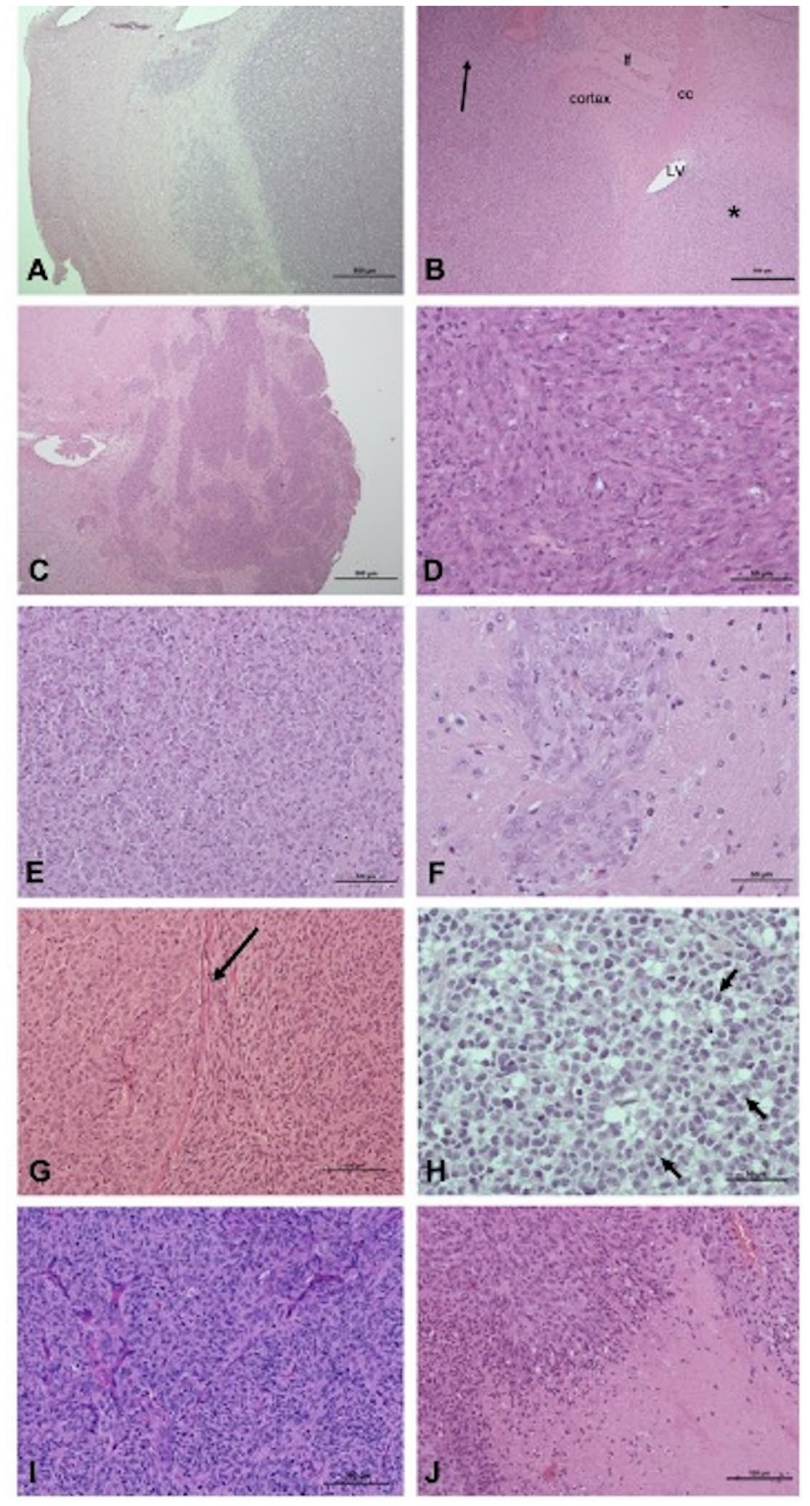
Pathologic features of xenografts: growth patterns **(A–C)**, cell morphology and architectural patterns **(D–H)**. **(A)** Focal infiltration, tumor G06A (H&E, scale bar: 500 μm): the interface between the tumor and the adjacent parenchyma, albeit readily identifiable, is multifocally infiltrated. **(B)** Diffuse infiltration, tumor J3T (H&E, scale bar: 500 μm): a diffuse neoplastic infiltrate is present within the caudate putamen (star) parietal cortex and leptomeninges (arrow). cc, corpus callosum; lf, longitudinal fissure of the brain; LV, lateral ventricle. **(C)** Diffuse infiltration associated with perivascular cuffing, J3T-Bg tumor (H&E, scale bar: 500 μm): coalescence of large perivascular cuffs forms a multinodular and poorly delineated mass. **(D)** J3T-Bg tumor (H&E, scale bar: 50 μm): spindle cells organized in fascicles. **(E)** J3T tumor (H&E, scale bar: 100 μm): polygonal cells organized in packets separated by a fine capillary network. **(F)** Tumor J3T-Bg (H&E, scale bar: 50 μm): polygonal cells organized in perivascular cuffs. **(G)** J3T tumor (H&E, scale bar: 100 μm): coexistence of a polygonal cell area and a spindle cell area separated from each other by the corpus callosum (arrow). **(H)** Tumor G06A (H&E, scale bar: 50 μm): sheets of round to polygonal cells with hyperchromatic nucleus and a pale perinuclear halo, associated with nuclear rowing (arrows). **(I)** J3T tumor (H&E, scale bar: 100 μm): numerous branching thin-walled capillaries within the tumor core. **(J)** J3T tumor (H&E, scale bar: 100 μm): geographic necrosis with palisading at the edges.

Histological examination of xenografts are displayed in Figure 3 and revealed densely cellular neoplasms with different growth patterns defined as: (i) focal infiltration (well-demarcated neoplasms with focal or multifocal regions of infiltration) for 100% of SDT3- and G06-derived xenografts and 30% of J3T-derived xenografts, (ii) diffuse infiltration (poorly demarcated neoplasms with a significant infiltration of the surrounding parenchyma and/or leptomeninges) for 10% of J3T-derived xenografts and (iii) diffuse infiltration associated with striking perivascular cuffing for 60% of J3T tumors and 100% of Raffray and J3T-Bg derived xenografts (Figures 3A–C). In J3T-Bg tumors, perivascular cuffs were large and coalescent leading to the formation of a multinodular mass at the injection site (Figure 3C). With regard to architectural patterns, cell morphology, cellular atypia and mitotic rate, all the xenografts were morphologically consistent with diffuse gliomas. G06A xenografts exhibited features of high-grade oligodendrogliomas. They were composed of sheets of round to polygonal cells with a central nucleus, varying from round and hyperchromatic to irregular with loose chromatin, surrounded by a moderately abundant pale cytoplasm (Figure 3H). The mitotic rate was comprised between 1 and 3 mitoses per high-power-field. Xenografts derived from J3T, J3T-Bg, SDT3 and Raffray cells were suggestive of an astrocytic differentiation. Their core was composed of either (i) interlacing or parallel fascicles of eosinophilic spindle cells with elongated nucleus, (ii) sheets and small packets of large polygonal cells with a prominent vesicular nucleus, and (iii) a combination of both. Tumor cells were also either spindle-shaped or polygonal in perivascular cuffs (Figures 3D–G). These xenografts were assigned as high-grade tumors on the basis of the mitotic rate and the presence of geographic necrosis with peripheric palisading in two J3T, two J3T-Bg and one Raffray-derived tumors (Figure 3J). Numerous branching thin-walled capillary vessels were consistently seen in the tumors but microvascular proliferation could not be observed in any of the samples (Figure 3I).

**Figure 4:**
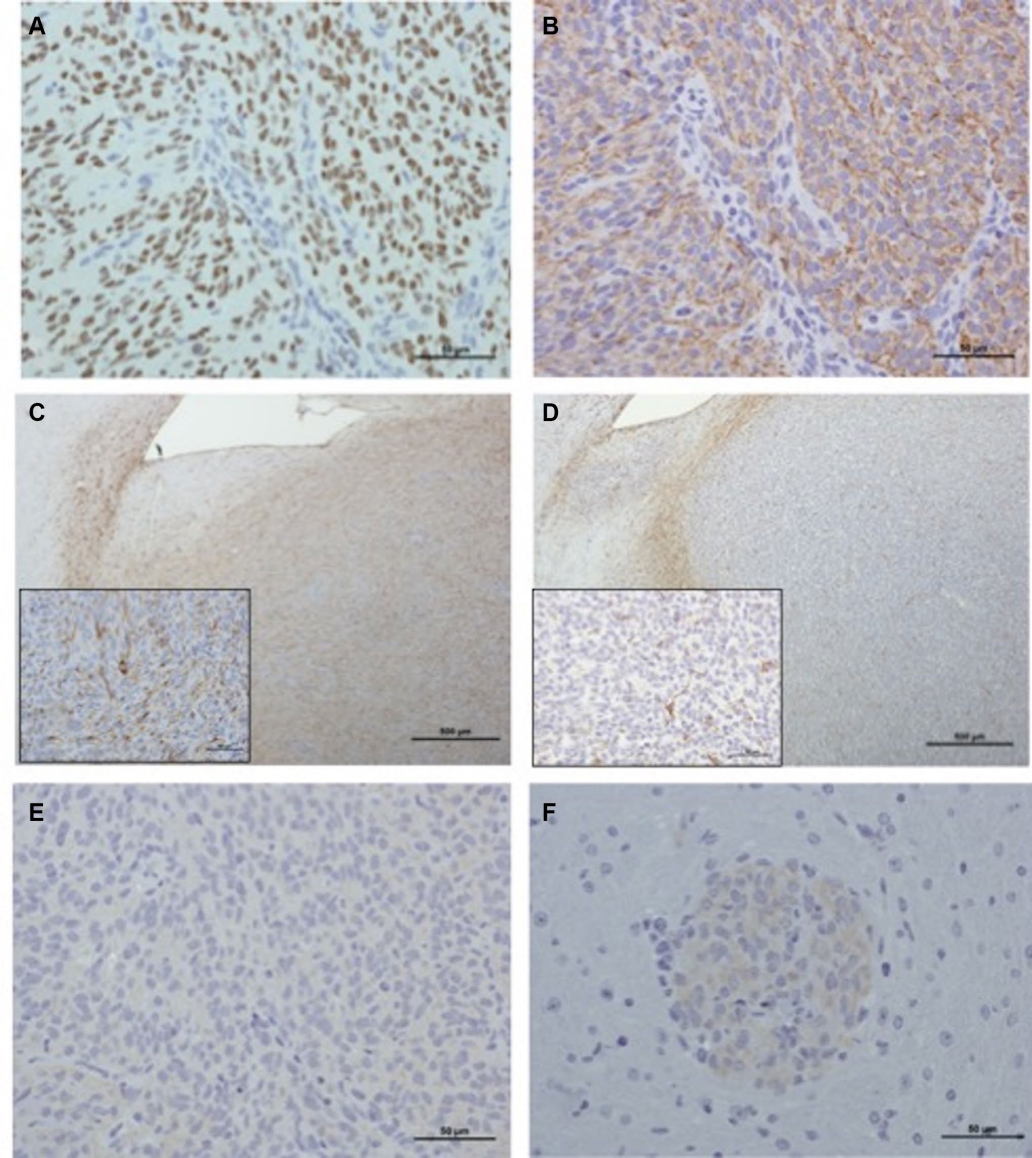
Immunohistochemical characterization of xenografts. **(A)** Raffray tumor (IHC Olig2, scale bar: 50 μm): nuclear staining is intense in more than 90% of the tumor population (IHC score: ++++). **(B)** Raffray tumor, same tumor area as shown in panel **(A)** (IHC GFAP, scale bar: 50 μm): 60 to 90% of the cells exhibit a cytoplasmic staining of medium intensity (IHC score: ++). **(C)** SDT3 tumor (IHC GFAP, scale bar: 500 μm): in this focally infiltrative tumor, GFAP labeling can be seen at the interface between tumor and adjacent parenchyma, but also in the tumor core where 30 to 60% of the cells are positive with a moderately intense finely granular cytoplasmic labeling (IHC score: ++). Inset (scale bar: 50 μm): a labeled mitotic tumor cell is present in the center of the image. **(D)** G06A (IHC GFAP, scale bar: 500 μm): in this focally infiltrative tumor, GFAP labeling can only be seen at the interface between tumor and adjacent parenchyma and corresponds to immunostaining of reactive normal astrocytes. There is no labeling of cells within the tumor core (inset scale bar: 50 μm), few normal astrocyte processes interspersed between neoplastic cells are labeled (IHC score: 0). **(E)** Raffray tumor, same tumor area as in panels **(A,B)** (IHC Vimentin, scale bar: 50 μm): tumor cells are negative to Vimentin (IHC score: 0). **(F)** J3T-Bg (IHC Vimentin, scale bar: 50 μm): perivascular cuffs exhibit a weak cytoplasmic immunolabeling (IHC score: +).

The results of immunohistochemical results obtained for the different xenografts are displayed in Supplementary Table S2 and Figure 4. Regarding J3T, SDT3, Raffray and G06-A-derived xenografts, Olig2 immunostaining confirmed the diagnosis of diffuse gliomas as 79% of these tumors exhibited a high IHC score for this marker. The absence of expression of the NeuN marker in these tumors corroborated their glial differentiation. All the J3T, SDT3 and Raffray-derived tumors were positive to GFAP with a medium IHC score, confirming their astrocytic origin. The absence of GFAP immunolabeling in G06A xenografts (Figure 4D) was consistent with the oligodendroglial phenotype observed in H&E-stained sections. The value of the glial marker S100 in the characterization of xenografts appeared limited in our study as the glial tumors with a high IHC score for Olig2 exhibited a weak or absent S100 immunostaining. Neoplastic cells did not express Vimentin in any of the tumor cores except that of J3T xenografts, but a weak immunopositivity to this marker was observed in J3T, Raffray and J3T-Bg perivascular cuffs. CD133 immunolabeling was negative in all the samples analyzed. Regarding J3T-Bg-derived xenografts, the tumor core did not express any of the markers; the only observable immunostaining was that of Vimentin in perivascular cuffs (Figure 4F). Despite the absence of Olig2 labeling, J3TBg tumors were assigned as diffuse gliomas considering their close morphology with J3T tumors (confirmed to be high-grade astrocytomas) and the presence of typical palisading necrotic areas in 2 tumors. Because of their lack of immunopositivity to different glial markers, they were considered to be poorly differentiated gliomas.

## 4 Discussion

The aim of the present study was to evaluate the intrinsic radiation sensitivity of five canine high-grade glioma (HGG) cell lines by performing clonogenic assays and examining the SF2 value and MID. To our knowledge, tumorigenicity and intrinsic radiosensitivity of canine HGG has never been assessed.

Before assessing their radiosensitivity, the 5 cell lines were confirmed to be neoplastic (cells exhibiting chromosomal abnormalities and severe cytonuclear atypia), astrocytic (cells immunopositive to Vimentin and GFAP and immunonegative to neuronal markers), canine in origin according to the karyotypic analysis and able to give rise to colonies according to the colony forming efficiency results. Our phenotypic results are in agreement with the literature relative to J3T and Raffray cells. As described in the study of Berens et al., we observed GFAP immunolabeling of more than 90% of J3T cells (37). In accordance with the study of Monod, Raffray cells demonstrated a weak and infrequent immunopositivity to GFAP associated with a strong Vimentin immunostaining of more than 90% of cells (36). Although the parent tumor of Raffray cells was diagnosed as a grade III oligodendroglioma, the phenotypic characteristics of the cultured cells suggest an astrocytic differentiation. The doubling times of canine glioma cells were found to be similar to those described in the literature for human GBM cell lines (from 24 to 60 h). The doubling time of J3T was shorter in our study compared to that reported in the literature (20.6 versus 49 in Berens’ study and 28 in Rainov’s study).

The radiosensitivity of canine cell lines was evaluated with a clonogenic assay. This test, which is the most used for the evaluation of the response to ionizing radiation, provide experimental survival curves representing the survival fraction (SF) as a function of radiation dose (44). Although the ability of SF2 to predict the clinical response of human HGG to radiotherapy has not been demonstrated (45, 46), this parameter is widely used to compare the radiosensitivity of different cancer cell lines or to assess the radiosensitizing potential of new therapeutics *in vitro* (47, 48). As it takes into account the entire survival curve, MID is also a useful parameter for quantifying differences in survival curves between cell lines. In our study, the mean SF2 and the mean MID were 84% and 4.84 Gy, respectively. However, SF2 values we obtained in this study are similar for J3T cell lines, higher for J3T-Bg and G06A compared to another recent study (49). These differences could be explained by the different culture medium between our two studies which could impact intrinsic radiosensitivity of cell lines. These values are higher than those described in the literature for human HGG cell lines (45). Indeed, the study of Taghian et al. evaluated SF2 and MID of 21 human HGG cell lines and reported a mean SF2 of 51% and a mean MID of 2.57 Gy. In this latter study, a wide variation in SF2 was observed between cell lines: some were obviously radioresistant (highest SF2 values between 70 and 80%) while others exhibited moderate radioresponsiveness (lowest SF2 values between 20 and 40%). Such a variation in SF2 has not been found in our study, maybe because of the limited number of cell lines of canine HGG. From various sarcomas and carcinomas, in the study of Maeda et al. for which the response of 27 canine cell lines to RT was evaluated, SF2 values as high as those of our study were described for mammary carcinoma and various sarcomas (50). The alpha and beta parameters provide information on the response of tumor cells to lethal and sublethal damages. Relevant information regarding the way to optimize RT treatment planning can also be provided by these parameters as the α/β ratio reflects the fractionation sensitivity of cells. For human HGG cell lines, the literature data indicate intermediate α/βvalues (5–10 Gy) (45, 51) and a mean alpha around 0.3 (52). By contrast with our study, the mean alpha/beta ratio and the mean alpha are, respectively, 0.836 Gy and 0.027 Gy^−1^. Such low values are only found in human prostastic cancers and malignant melanoma (53, 54). According to the quadratic model, tumors exhibiting an α/β ratio under 2 Gy are more sensitive to hypofractionation. Our results are in agreement with the results of a recent study showing that a hypofractionated radiation protocol performed in dogs for the treatment of intracranial tumors (10 × 4 Gy) leads to the same overall survival as the classical approach (20 × 2.5 Gy) without additional side effects.

The intracerebral implantation of the five canine cell lines available to us in the laboratory has confirmed their ability to reproduce high-grade gliomas of malignancy when they are implanted orthotopically: astrocytomas for J3T, SDT3 and Raffray, oligodendroglioma for G06A and poorly differentiated glioma for J3T-Bg. We confirmed that these cells lines were diffuse high-grade glioma as they were all positive for Olig2 except for J3T-Bg cell lines, and astrocytic type for those positive for GFAP and Vimentine (55). In addition of Olig2, CNPase marker could be used with G06A cell lines to confirm the oligodendroglial differentiation of this cell lines (14, 56). The xenograft phenotype is consistent with that of the original tumor for J3T cells (from anaplastic astrocytoma) and SDT3 (from glioblastoma). The very little differentiated character of J3T-Bg can be explained by the fact that these cells were recovered after subcutaneous implantation of J3T cells in Biege Nude XID mice, this passage potentially having led to the selection of a cell subpopulation (16). The Raffray cells were isolated from a grade III oligodendroglioma. *In vitro* analysis of the expression profile of the cell line carried out by the laboratory that isolated them revealed a poorly differentiated astrocytic phenotype, a phenotype confirmed by the analysis we carried out on these cells included in HistoGel. The orthotopic implantation of Raffray therefore confirms what had been observed *in vitro*: the isolated tumor cells reproduce a high-grade astrocytoma and not an oligodendroglioma, which seems to indicate a selection of astrocytic tumor cells during the establishment of the cell line. Finally, the tumors obtained from the G06A do not reproduce the original tumor both in terms of phenotype (the xenograft is oligodendroglial, the tumor of astrocytic origin) and of grade of malignancy (grade III for the xenograft, grade IV for the original tumor). This difference between the original tumor and xenograft for G06A and Raffray could be explained by intrinsic plasticity of glial tumors, leading to slight modifications in tumor phenotype and markers expression between cell culture and xenograft (57). That drift for G06A and Raffray from rate of engraftment of G06A cells (only 25%) and the phenotype that they induce when injected make them the least interesting cell line to use in a perspective of future implementation of a model of murine study of canine diffuse glioma.

There is growing evidence that pet dogs with spontaneous HGG are relevant models for studying human diffuse gliomas (8, 10). These dogs therefore appear as potential animal models for preclinical testing of novel treatment strategies. Here, we achieved a radiobiological characterization of five canine HGG cell lines (J3T, J3T-Bg, SDT3, G06A and Raffray). Their general characteristics and their ability to generate tumors in immunocompromised mice were also evaluated. Among these five canine HGG cell lines, J3T, J3T-Bg and SDT3 validate the value of the canine glioma model for further research.

## Data availability statement

The raw data supporting the conclusions of this article will be made available by the authors, without undue reservation.

## Ethics statement

The animal study was approved by Institution animal ethics committee with authorization number 2018031614514282. The study was conducted in accordance with the local legislation and institutional requirements.

## Author contributions

BC and AD: writing – original draft, conceptualization, and methodology. CD: conceptualization and resources. JA: resources. MP: conceptualization, resources, and supervision. EC-J and GM: supervision. All authors contributed to the article and approved the submitted version.
