# Supplementary material for: Evaluation of in vitro intrinsic radiosensitivity and characterization of five canine high-grade glioma cell lines
Source: Front Vet Sci. 2023 Nov 30;10:1253074. doi: 10.3389/fvets.2023.1253074 (PMC10720585; doi:10.3389/fvets.2023.1253074)
Supplement: Supplementary file 1 [file Data_Sheet_1.pdf]

Supplementary table 1 : *Immunohistochemical markers used for the study of HistoGel-encapsulated cells and/or excised brains of mice*

	**Antibody name**	**Company**	**Dilution**	**Pretreatment**	**Specimen**
**CD133**	Rabbit Polyclonal to CD133 stem cell marker	Abcam 19898	1:200	Citrate buffer 0.01M pH 6, 20’ water bath + 30’ room temperature.	HistoGel-encapsulated cells and excised brains
**DCx**	Rabbit Polyclonal to Duble cortin neuronal marker	Abcam ab18723	1:1000	Citrate buffer 0.01M pH 6, 20’ water bath + 30’ room temperature.	HistoGel-encapsulated cells
**ßIII**	Mouse monoclonal anti-tubulin ßIII isoform	Millipore MAB1637	1:200	Citrate buffer 0.01M pH 6, 20’ water bath + 30’ room temperature.	HistoGel-encapsulated cells
**NeuN**	Mouse anti neuronal nuclei	Chemicom MAB377	1:500	Citrate buffer 0.01M pH 6, 20’ water bath + 30’ room temperature.	HistoGel-encapsulated cells and excised brains
**Olig2**	Rabbit anti Olig 2 Polyclonal Antibody	Millipore (Chemicon) AB 9610	1:100	Citrate buffer 0.01M pH 6, 20’ water bath + 30’ room temperature.	HistoGel-encapsulated cells and excised brains
**GFAP**	Rabbit Anti-Cow Glial Fibrillary Acidic Protein	Dako Z0334	1:500	Citrate buffer 0.01M pH 6, 20’ water bath + 30’ room temperature.	HistoGel-encapsulated cells and excised brains
**S-100**	Rabbit anti S-100	Dako Z0311	1:1000	Citrate buffer 0.01M pH 6, 20’ water bath + 30’ room temperature.	Excised brains
**VIM**	Mouse Monoclonal anti vimentin clone V9	Dako M0725	1:200	Without pretreatment	HistoGel-encapsulated cells and excised brains

Supplementary table 2 : *Immunohistochemical features of xenografts. IHC: Immunohistochemical. Neg: negative. (*): In 7/9 J3T-Bg tumors and 2/3 Raffray tumors, a cytoplasmic and diffuse Olig2 staining was observed within perivascular cuffs but was considered negative in the absence of a concomitant nuclear labeling*

	J3T		J3T-Bg		SDT3	G06	Raffray	
	Tumor core	Perivascular cuffs	Tumor core	Perivascular cuffs	Tumor core	Tumor core	Tumor core	Perivascular cuffs
**Olig2**								
Number of xenografts analyzed	9	6	9	9	8	2	4	3
Number of positive xenografts	9	6	0	0*	8	2	3	0*
**Mean IHC score**	**+++**	**+**	**Neg**	**+**	**+++**	**++++**	**+++**	**+**
**GFAP**								
Number of xenografts analyzed	2	2	2	2	2	2	2	2
Number of positive xenografts	2	2	0	0	2	0	2	1
**Mean IHC score**	**+++**	**++**	**Neg**	**Neg**	**++**	**Neg**	**++**	**+**
**Vimentine**								
Number of xenografts analyzed	2	2	2	2	2	2	2	2
Number of positive xenografts	0	2	0	2	1	0	1	2
**Mean IHC score**	**Neg**	**++++**	**Neg**	**+**	**Neg**	**Neg**	**Neg**	**+**
S100								
Number of xenografts analyzed	2	2	2	2	2	2	2	2
Number of positive xenografts	2	1	0	0	2	0	2	1
**Mean IHC score**	**+**	**++**	**Neg**	**Neg**	**+**	**Neg**	**++**	**+**
**NeuN**								
Number of xenografts analyzed	2	2	2	2	2	2	2	2
Number of positive xenografts	0	0	0	0	0	0	0	0
**Mean IHC score**	**Neg**	**Neg**	**Neg**	**Neg**	**Neg**	**Neg**	**Neg**	**Neg**
**CD133**								
Number of xenografts analyzed	2	2	2	2	2	2	2	2
Number of positive xenografts	0	0	0	2	0	0	0	0
**Mean IHC score**	**Neg**	**Neg**	**Neg**	**+**	**Neg**	**Neg**	**Neg**	**Neg**
